# Integrity of hypothalamic–pituitary‐testicular axis in exceptional longevity

**DOI:** 10.1111/acel.13656

**Published:** 2022-06-29

**Authors:** Sandra Aleksic, Dimpi Desai, Kenny Ye, Sally Duran, Tina Gao, Jill Crandall, Gil Atzmon, Nir Barzilai, Sofiya Milman

**Affiliations:** ^1^ Department of Medicine, Division of Endocrinology, Institute for Aging Research Albert Einstein College of Medicine Bronx New York USA; ^2^ Department of Medicine, Division of Endocrinology Baylor College of Medicine Houston Texas USA; ^3^ Department of Epidemiology and Population Health (Biostatistics) Albert Einstein College of Medicine Bronx New York USA; ^4^ Department of Systems & Computational Biology Albert Einstein College of Medicine Bronx New York USA; ^5^ Department of Genetics Albert Einstein College of Medicine Bronx New York USA; ^6^ Department of Natural Science University of Haifa Haifa Israel

**Keywords:** centenarians, endocrinology, human, hypothalamus, longevity, testosterone

## Abstract

Hypothalamic integrity increasingly is being recognized as a marker of healthy longevity in rodent models. Insight into hypothalamic function in humans with exceptional longevity can be gained via investigation of the hypothalamic–pituitary‐testicular (HPT) axis in men with exceptional longevity. This study aimed to characterize the HPT axis function, defined by levels of testosterone (T) and luteinizing hormone (LH), in 84 Ashkenazi Jewish men aged 90–106 years. We found that 94% of men exhibited preserved hypothalamic–pituitary function, as evidenced by either normal testosterone and LH levels (25%) or an appropriate rise in LH in response to aging‐related primary testicular dysfunction (69%), a hormone pattern mirroring female menopause. Total T level was not associated with metabolic parameters or survival. These results demonstrate a high prevalence of testicular dysfunction with preserved hypothalamic–pituitary function in men with exceptional longevity. Thus, the role of hypothalamic integrity and HPT axis in healthy aging warrants further investigation.

## Graphical abstract

The hypothalamus is increasingly being recognized as a key player in longevity. Hypothalamic integrity in men with longevity can be inferred from the function of the hypothalamic‐pituitary‐testicular (HPT) axis. We herein found that the majority of nonagenarian and centenarian men have preserved hypothalamic regulation of the HPT axis.

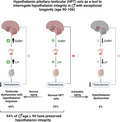

## 1 INTRODUCTION, RESULTS AND DISCUSSION

Hypothalamic dysfunction increasingly is recognized as an important contributor to systemic aging (Chellappa et al., 2019; Debarba et al., 2022; Sadagurski et al., 2017; Sadagurski, Landeryou, Cady, Bartke, et al., 2015; Sadagurski, Landeryou, Cady, Kopchick, et al., 2015; Wang et al., 2021; Zhang et al., 2013; Zhang et al., 2017). In male mice, systemic aging was accelerated by dysregulated hypothalamic secretion of gonadotropin releasing hormone (GnRH) (Wang et al., 2021). Hypothalamic GnRH controls the hypothalamic–pituitary‐testicular (HPT) axis, whereby GnRH secreted from the hypothalamus stimulates the pituitary gland to make luteinizing hormone (LH), which in turn stimulates the testes to produce testosterone (T). T acts on the hypothalamus and pituitary via negative feedback to inhibit GnRH and LH production, respectively (Handelsman, 2000). A drop in T results in loss of negative feedback and elevations in GnRH and LH. Although hypothalamic GnRH cannot be measured in humans directly, due to its pulsatile secretion and low circulating levels, its production can be inferred from measurements of circulating LH and T. Low T level that is not accompanied by compensatory secretion of GnRH and LH (Tajar et al., 2010) is indicative of hypothalamic dysfunction. In contrast, a compensatory rise in LH in response to age‐related testicular dysfunction is indicative of preserved hypothalamic function. This compensatory hypothalamic–pituitary response may be sufficient to maintain normal T levels in men with age‐related testicular dysfunction, manifesting as compensated testicular dysfunction, or may be insufficient, resulting in overt testicular dysfunction, similar to female menopause (Tajar et al., 2010). Studying the integrity of the HPT axis in men with exceptional longevity, who delay the onset of age‐related diseases, can offer insight into the role that the hypothalamus plays in resilience to aging in humans. We hypothesized that healthy longevity in men will be associated with a preserved hypothalamic response to age‐related testicular dysfunction. Thus, we performed the largest study till date that evaluated the integrity of the HPT axis in nonagenarian and centenarian men.

**FIGURE 1:**
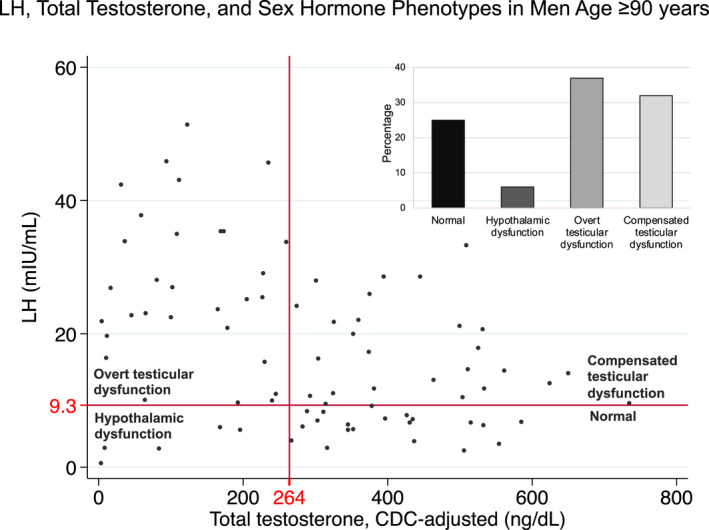
LH, TT levels and sex hormone phenotypes in men age ≥ 90 years (*n* = 81)

The study included 84 Ashkenazi Jewish men, age 90–106 years, from the Longevity Genes Project cohort (Atzmon et al., 2004); Table 1), with available sera collected at enrolment. Measurements included total testosterone (TT) by LC/MS, calculated free T (Vermeulen et al., 1999), LH and sex‐hormone binding globulin (SHBG). In the results that follow (Figure 1), low TT level was defined as Centers for Disease Control (CDC)‐adjusted TT level (Travison et al., 2017; Appendix S1) below the 2.5th percentile for CDC‐harmonized levels for young men (264 ng/dl) (Bhasin et al., 2018). Elevated LH levels were defined based on upper reference limit (Tajar et al., 2010) provided by Quest Laboratories (9.3 mIU/ml). Only 6% of men had evidence of hypothalamic dysfunction (low TT and non‐elevated LH). The remainder of men had hormonal patterns consistent with normal hypothalamic regulation of the HPT axis. Twenty‐five percent had normal TT and LH levels while the rest of the men demonstrated preserved hypothalamic response to testicular dysfunction: 37% had overt testicular dysfunction (low TT and elevated LH) and 32% had testicular dysfunction that was compensated by an increased hypothalamic–pituitary response (normal TT and elevated LH). To discern between longevity vs. end‐of‐life phenotypes, we performed a sensitivity analysis by excluding men who died within 1 year of enrolment and found a similar distribution of sex hormone phenotypes (Table S1).

**TABLE 1:** Participant characteristics (*n* = 84)

Age, years	97.2 ± 3.1
Community dwelling, %	67
BMI, kg/m^2^ (*n* = 72)	23.4 ± 3.0
Total testosterone, ng/dl	279 ± 176
CDC‐adjusted to5tal testosterone, ng/dl	287 ± 181
Free testosterone, ng/dl	3.3 ± 2.1
LH, mIU/ml (*n* = 81)	14.7 (7.3–25.5)
SHBG, nmol/L	72 ± 23
Total cholesterol, mg/dl (*n* = 82)	174 ± 42
Triglycerides, mg/dl (*n* = 82)	130 ± 66
LDL cholesterol, mg/dl (*n* = 82)	98 ± 36
HDL cholesterol, mg/dl (*n* = 82)	50 ± 14
Random plasma glucose, mg/dl (*n* = 83)	100 ± 2

*Note*: Data are mean ± SD, except for LH (median [IQR]). Number in parenthesis (*n*) indicates men with available data when data are missing.

Multivariable linear regression model that included age and a priori selected metabolic variables did not show significant associations between TT levels and body mass index (BMI) (*p* = 0.83), serum triglycerides (*p* = 0.87), HDL cholesterol (*p* = 0.78), LDL cholesterol (*p* = 0.20) or random glucose levels (*p* = 0.59) (Table S2). Adjustment for SHBG, a carrier protein for T whose concentrations increase with age (Yeap et al., 2007), did not meaningfully affect the associations (Table S3). Cox proportional hazard analysis in men with known vital status (*n* = 78) demonstrated that TT was not statistically significantly associated with survival after adjusting for age (HR = 1.00, 95% CI: 0.87–1.17, per 100 ng/dl difference in TT).

Evidence from male rodents (Gruenewald et al., 2000; Wang et al., 2021) and men (Takahashi et al., 2005) demonstrates that aging is associated with dysregulation of hypothalamic GnRH pulses. While it remains challenging to directly evaluate disruption of hypothalamic GnRH pulses in humans, there is abundant epidemiologic evidence that metabolically unhealthy aging, characterized by obesity (Travison et al., 2007) and diabetes mellitus (Dhindsa et al., 2010), exacerbates hypothalamic dysregulation of the HPT axis. Clinically, these changes manifest as low T without compensatory LH response, indicative of hypothalamic dysfunction and are present in 11% of men from the general population age 40–79 years (Tajar et al., 2010). By contrast, only 6% of men in this study exhibited the absence of hypothalamic response to low T, despite being markedly older than the previously studied cohorts (Tajar et al., 2010). A decline in T observed in men with exceptional longevity in our cohort predominantly resulted from testicular dysfunction, which has been attributed to the loss of Leydig cell mass (Neaves et al., 1984), reduced steroidogenic capacity (Luo et al., 2001) and impairments in testicular microenvironment (Curley et al., 2019). Our findings indicate that sex hormone patterns resembling menopause are common in men with exceptional longevity, but these changes occur decades later than in women.

Whether impaired testicular function with preserved hypothalamic response impacts health at extreme age is yet to be fully elucidated in longitudinal studies. Nonetheless, our results indicate that low TT may not be associated with unfavourable metabolic profile or mortality in men with exceptional longevity, who predominantly preserve the neuroendocrine response to age‐related testicular dysfunction. The role of the neuroendocrine system and its main regulator, the hypothalamus, in healthy aging is being increasingly recognized. In rodents, hypothalamic inflammation and suppressed GnRH secretion resulted in accelerated aging, whereas inhibition of hypothalamic inflammation and treatment with GnRH delayed aging (Zhang et al., 2013). In humans, disruptions in structural integrity of the hypothalamus have been implicated in insulin resistance (Schur et al., 2015), obesity (Thomas et al., 2019) and male hypogonadism (Berkseth et al., 2018).

Extrapolation of our findings to the general population is limited by survivor bias; however, the focus of this study was on the phenotype of exceptional longevity. Observed sex hormone phenotypes may be a feature of exceptional longevity or a harbinger of impending death; however, we observed similar distribution of sex hormone phenotypes when men who died in the first year of follow‐up were excluded, suggesting that the observed phenotypes are features of longevity. While experimental dynamic testing may provide more nuanced assessment of hypothalamic function, such invasive testing would not be practical in men with exceptional longevity; therefore, we used LH response to testicular dysfunction as a surrogate of hypothalamic integrity, as has been established in clinical practice. Although the samples were not collected in fasted state or early morning, the impact on results is likely modest since circadian rhythm (Bremner et al., 1983) and prandial fluctuations (Van de Velde et al., 2020) of T secretion are attenuated in older men. Furthermore, LH does not display significant circadian variation (Brambilla et al., 2009); thus, the rise in LH coupled with reduced T levels was indicative of true low T with reciprocal hypothalamic–pituitary compensation. A major strength of the study is that it included the largest number of nonagenarians and centenarians of any study to date that evaluated the HPT axis, thus providing a window into the integrity of the HPT axis in exceptionally long‐lived men.

In conclusion, our study demonstrated a high prevalence of testicular dysfunction accompanied by a preserved hypothalamic–pituitary response in men with exceptional longevity, indicative of preserved hypothalamic integrity. Future longitudinal studies are warranted to establish the role of the HPT axis as a biomarker for healthy longevity.

## AUTHOR CONTRIBUTIONS

JC, NB and SM designed the study. DD, SD, TG, GA and SM performed data acquisition. SA, KY and SM analyzed the data. SA, DD, KY, SD, TG, JC, GA, NB and SM performed writing and critically reviewing the manuscript.

## CONFLICT OF INTEREST

None declared.

## Supporting information


Appendix S1

## Data Availability

The data that support the findings of this study are available on request from the corresponding author. The data are not publicly available due to privacy or ethical restrictions.
