# Supplementary material for: Integrity of hypothalamic–pituitary‐testicular axis in exceptional longevity
Source: Aging Cell. 2022 Jun 29;21(8):e13656. doi: 10.1111/acel.13656 (PMC9381897; doi:10.1111/acel.13656)
Supplement: Supplementary file 1 — Appendix S1 [file ACEL-21-e13656-s001.docx]

**Male Menopause Reveals Integrity of Hypothalamic-Pituitary-Testicular Axis in Exceptional Longevity**

**Supplementary Information**

**Appendix S1. Subjects and Methods:**

*Study population:*

The study included men from the cross-sectional Longevity Genes Project (LGP) cohort established at the Albert Einstein College of Medicine (Einstein)^1^. The LGP cohort enrolled Ashkenazi Jewish individuals who reached at least 95 years of age, their offspring, and controls without parental longevity, who were sex- and age- matched to the offspring. Men aged ≥90 years recruited into the LGP study between 1998 and 2014 who had a stored serum sample available were eligible for this study (n = 85). One man who was actively treated with testosterone was excluded from the analysis (n = 1). No eligible men were actively treated with GnRH agonists. Vital status was determined as of December 4, 2020, through reports of the next of kin or review of publicly available data, with 75 (89%) confirmed deaths, 3 (4%) confirmed alive at last time of contact, and 6 (7%) lost to follow-up after the enrollment. Study physical assessments included weight and height. A venous blood sample in all participants was collected in the morning or the early afternoon under non-fasting conditions; the processed serum and plasma were stored at -80°C. Written informed consent was obtained from all participants or their proxies. The study was approved by Einstein’s Institutional Review Board.

*Laboratory measures:*

Total testosterone was measured using LC-MS (Thermo Fisher Quantum Ultra mass spectrometer and Thermo Fisher Aria LC) at the Quest Diagnostics Nichols Institute Laboratories (Quest) in Valencia, CA. For total testosterone, the lowest limit of detection (LOD) was 0.84 ng/dL (0.03 nmol/L) and the coefficient of variance (CV) ranged from 9.94% to 3.05% for the very low (11.64 ng/dL [0.40 nmol/L]) to high (1073.54 ng/dL [37.25 nmol/L]) controls, respectively. Quest’s total testosterone assay is certified by the Centers for Disease Control (CDC) Hormone Standardization (HoSt) Program. During the time period when total testosterone was measured (April 29-30, 2015), the mean bias per the CDC HoSt program for male samples was −3.9% (95% Confidence Interval [CI]: −5.5% to −2.4%). Being that no changes in bias were observed with testosterone reference values (i.e. there were no trends in bias over concentration), a single factor correction was used to adjust measured testosterone values to the CDC reference method with the following formula: *CDC-adjusted testosterone level = (measured testosterone level x 100) / 97.1.* Free testosterone was calculated according to Vermeulen et al.^2^. In 73 participants with missing serum albumin values, an albumin value of 4.3 g/dL was used to calculate free testosterone^2^.

SHBG and LH were measured using immunoassays (Beckman DXI and Siemens Centaur, respectively) at Quest. For the SHBG assay the LOD was 2 nmol/L and the CV was 6.25%. The LOD for the LH assay was 0.2 mIU/mL (0.2 IU/L) and the CV was 5%. Serum total cholesterol, triglycerides, and high-density lipoprotein (HDL) cholesterol were measured by standard automated methods. The Friedwald formula was used to calculate low-density lipoprotein (LDL) cholesterol levels^3^.

Low testosterone levels were defined using the 2.5^th^ percentile value for young, non-obese men (264 ng/dL) for CDC-harmonized total testosterone^4,5^ that were applied to CDC-adjusted total testosterone values in our cohort. Elevated LH levels were defined based on upper reference limit for men <60 years provided by Quest (9.3 mIU/mL). Sex hormone phenotypes were defined as follows: 1) normal: CDC-adjusted total testosterone level at or above 264 ng/dL and LH level at or below 9.3 mIU/mL; 2) testicular dysfunction: testosterone level below 264 ng/dL and LH above 9.3 mIU/mL; 3) compensated testicular dysfunction: testosterone level at or above 264 ng/dL and LH above 9.3 mIU/mL; and 4) hypothalamic dysfunction: testosterone level below 264 ng/dL and LH at or below 9.3 mIU/mL.

*Statistical analysis:*

Normality was assessed by inspection of the histograms. Parametric data is presented as mean ± standard deviation (SD) and nonparametric data as median (interquartile range).

Multivariable regression analysis

Given the known association between obesity and metabolic dysfunction and the most common hormone pattern in older men with low testosterone, hypothalamic dysfunction^6^, we investigated the associations between metabolic dysfunction and total testosterone levels. We performed multivariable linear regression analysis using *a priori* selected metabolic variables (body mass index [BMI], random serum glucose, serum triglycerides, HDL, and LDL cholesterol) and age as independent predictors, and total testosterone levels as the dependent variable. Sensitivity analysis included model that was additionally adjusted for SHBG. Regression diagnostics did not reveal violations in assumptions of residual normality, homoskedasticity, linearity, or presence of multicollinearity or meaningfully influential observations.

Survival analysis

Cox proportional hazard model was used to assess whether total testosterone level, as a continuous variable, was associated with risk of mortality, with age at enrollment as a covariate. Individuals who did not die were censored at the date they were last confirmed to be alive. Proportional hazards assumption was confirmed by assessment of Schoenfeld residuals, including the inspection of plots against log time and statistical testing.

Statistical analysis was performed using STATA software, version 15 (StataCorp LP, College Station, TX). A two-tailed p-value <0.05 was considered statistically significant.

**Supplementary Tables**

**Table S1. Sex hormone phenotypes in men with survival ≥ 1 year (n=60).**

| **Phenotype** |  | **N (%)** |
| --- | --- | --- |
| **Normal** |  | 15 (25) |
| **Hypothalamic dysfunction** |  | 3 (5) |
| **Overt Testicular Dysfunction** |  | 22 (37) |
| **Compensated Testicular Dysfunction** |  | 20 (33) |

Out of 84 men, 16 men had survival shorter than one year, 6 men had missing data for survival and another two men had missing data for LH levels (thus sex hormone phenotype could not be determined).

**Table S2. Adjusted beta coefficients for metabolic predictors of total testosterone levels in men with available data (n = 70).**

| **Covariate** |  | **Beta** | **95% CI** | **p-value** |
| --- | --- | --- | --- | --- |
| **Age** |  | −8.1 | −22.6, 6.3 | 0.27 |
| **BMI** |  | 1.6 | −13.6, 16.9 | 0.83 |
| **Triglycerides** |  | 0.6 | −6.5, 7.7 | 0.87 |
| **HDL** |  | 4.4 | −26.4, 35.2 | 0.78 |
| **LDL** |  | 7.8 | −4.2, 19.9 | 0.20 |
| **Random Glucose** |  | 4.2 | −11.4, 19.7 | 0.59 |

*Beta coefficient for age is expressed per 1 year difference, for BMI per 1 kg/m^2^ difference, and per 10 mg/dL difference for triglycerides (0.113 mmol/L), HDL, LDL (0.259 mmol/L), and glucose (0.556 mmol/L). R^2^ = 0.07. BMI: Body mass index; HDL: High-density lipoprotein cholesterol; LDL: low-density lipoprotein cholesterol.

**Table S3. Adjusted beta coefficients for metabolic parameters and SHBG as predictors of total testosterone levels in men with available data (n = 70).**

| **Covariate** |  | **Beta** | **95% CI** | **p-value** |
| --- | --- | --- | --- | --- |
| **Age** |  | −8.5 | −22.5, 5.6 | 0.23 |
| **BMI** |  | 4.3 | −10.8, 19.4 | 0.57 |
| **Triglycerides** |  | 0.6 | −6.3, 7.6 | 0.85 |
| **HDL** |  | 0.9 | −29.3, 31.0 | 0.95 |
| **LDL** |  | 6.2 | −5.7, 18.0 | 0.30 |
| **Random Glucose** |  | 3.5 | −11.6, 18.6 | 0.65 |
| **SHBG** |  | 1.9 | 0.1, 3.8 | 0.04 |

*Beta coefficient for age is expressed per 1 year difference, for BMI per 1 kg/m^2^ difference, per 1nmol/L difference for SHBG, and per 10 mg/dL difference for triglycerides (0.113 mmol/L), HDL, LDL (0.259 mmol/L), and glucose (0.556 mmol/L). R^2^ = 0.13. BMI: Body mass index; HDL: High-density lipoprotein cholesterol; LDL: low-density lipoprotein cholesterol.

References

1. Atzmon G, Schechter C, Greiner W, Davidson D, Rennert G, Barzilai N. Clinical phenotype of families with longevity. Research Support, Non-U.S. Gov't

Research Support, U.S. Gov't, P.H.S. *Journal of the American Geriatrics Society*. Feb 2004;52(2):274-7.

2. Vermeulen A, Verdonck L, Kaufman JM. A critical evaluation of simple methods for the estimation of free testosterone in serum. *The Journal of clinical endocrinology and metabolism*. Oct 1999;84(10):3666-72. doi:10.1210/jcem.84.10.6079

3. Friedewald WT, Levy RI, Fredrickson DS. Estimation of the concentration of low-density lipoprotein cholesterol in plasma, without use of the preparative ultracentrifuge. *Clin Chem*. Jun 1972;18(6):499-502.

4. Travison TG, Vesper HW, Orwoll E, et al. Harmonized Reference Ranges for Circulating Testosterone Levels in Men of Four Cohort Studies in the United States and Europe. *J Clin Endocrinol Metab*. Apr 1 2017;102(4):1161-1173. doi:10.1210/jc.2016-2935

5. Bhasin S, Brito JP, Cunningham GR, et al. Testosterone Therapy in Men With Hypogonadism: An Endocrine Society Clinical Practice Guideline. *J Clin Endocrinol Metab*. May 1 2018;103(5):1715-1744. doi:10.1210/jc.2018-00229

6. Tajar A, Forti G, O'Neill TW, et al. Characteristics of secondary, primary, and compensated hypogonadism in aging men: evidence from the European Male Ageing Study. *J Clin Endocrinol Metab*. Apr 2010;95(4):1810-8. doi:10.1210/jc.2009-1796
